# Association of Pan-Immune-Inflammation Value with All-Cause and Cardiovascular Mortality in Survivors of Myocardial Infarction: NHANES 2001–2018 Analysis

**DOI:** 10.3390/jcdd12090363

**Published:** 2025-09-17

**Authors:** Qingyi Liu, Wenling Yang, Ruiyu Zhang, Xiaopeng Guo, Yumiao Wei

**Affiliations:** 1 Department of Cardiology, Union Hospital, Tongji Medical College, Huazhong University of Science and Technology, Wuhan 430022, China; m202376107@hust.edu.cn (Q.L.); d202382084@hust.edu.cn (W.Y.); m202476260@hust.edu.cn (R.Z.); 2 Hubei Key Laboratory of Biological Targeted Therapy, Union Hospital, Tongji Medical College, Huazhong University of Science and Technology, Wuhan 430022, China; 3 Hubei Provincial Engineering Research Center of Immunological Diagnosis and Therapy for Cardiovascular Diseases, Union Hospital, Tongji Medical College, Huazhong University of Science and Technology, Wuhan 430022, China; 4 Key Laboratory of Biological Targeted Therapy (Huazhong University of Science and Technology), Ministry of Education, Wuhan 430022, China; 5 Department of Radiology, Union Hospital, Tongji Medical College, Huazhong University of Science and Technology, Wuhan 430022, China

**Keywords:** pan-immune-inflammation value, all-cause mortality, cardiovascular mortality, myocardial infarction, NHANES

## Abstract

Background: Inflammatory responses critically impact long-term outcomes in myocardial infarction (MI) survivors, yet few biomarkers comprehensively evaluate systemic immune-inflammatory status. This study assessed the prognostic utility of a novel marker—the pan-immune-inflammation value (PIV)—for predicting all-cause and cardiovascular mortality post-MI. Methods: Using the National Health and Nutrition Examination Survey data (2001–2018), 1559 MI survivors were included. PIV was calculated as (neutrophils × platelets × monocytes)/lymphocytes. Weighted Cox models assessed the association between log-transformed PIV (LnPIV) and mortality. Restricted cubic spline (RCS) models explored non-linear dose–response relationships, and predictive performance was evaluated via time-dependent ROC analysis. Results: Over a median 75-month follow-up, 675 deaths occurred. LnPIV showed significant non-linear associations with all-cause (*p* < 0.0001) and cardiovascular mortality (*p* = 0.0471). When LnPIV ≥ 5.59, each unit increase was associated with an 85% (HR = 1.85, 95% CI: 1.49–2.28) higher all-cause mortality risk; for cardiovascular mortality, the risk increased by 77% (HR = 1.77, 95% CI: 1.20–2.63) when LnPIV ≥ 5.68. Time-dependent ROC analysis confirmed strong prediction above these thresholds. Conclusion: PIV demonstrates threshold-dependent mortality risk stratification in MI patients, particularly effective in high-inflammatory subgroups, offering a potential tool for personalized risk stratification.

## 1. Introduction

Myocardial infarction (MI) is one of the leading global causes of mortality and disability [1]. With advancements in modern diagnostic and therapeutic technologies, such as early reperfusion therapy, pharmacological interventions, and rehabilitation, the acute-phase survival rate of MI patients has significantly improved [2]. However, the long-term prognosis of survivors is influenced by multiple factors, including age, sex, comorbidities, cardiac functional status, and treatment modalities [3]. Additionally, the inflammatory response plays a crucial role in the pathophysiological processes following MI [4]. Post-infarction inflammation contributes to ventricular remodeling, heart failure, and other complications through various mechanisms, with inflammatory cells such as neutrophils, monocytes, and lymphocytes being central to this process [5].

The pan-immune-inflammation value (PIV) represents a novel composite indicator of systemic inflammation, derived from standard blood test components through the formula: (neutrophils × platelets × monocytes)/lymphocytes, this formula was first proposed by Fucà et al. in a cohort of patients with metastatic colorectal cancer, where PIV was shown to be independently associated with both overall survival and progression-free survival [6]. The rationale for including neutrophils, monocytes, and platelets in the numerator lies in their established roles as pro-inflammatory and pro-thrombotic mediators that contribute to tissue injury and disease progression [7,8], whereas lymphocytes are placed in the denominator because lymphopenia often reflects impaired adaptive immune function and reduced immunoregulatory capacity [9]. Consequently, a higher PIV captures the dual state of heightened inflammation/thrombosis and suppressed immune surveillance, making it a sensitive index of immune-inflammatory imbalance. Although PIV originated in oncology research [6], its biological rationale extends beyond cancer. Subsequent studies have demonstrated its prognostic value in a broad spectrum of diseases characterized by systemic inflammation, including autoimmune diseases [10] and cardiovascular diseases [11,12,13]. Given the intrinsic link between MI and inflammation, PIV holds promise as a marker for assessing inflammatory burden in MI survivors [14].

Preliminary studies have explored the clinical relevance of the PIV in MI. An observational study demonstrated that elevated PIV levels showed a significant correlation with impaired coronary flow (ICF) following percutaneous coronary intervention (PCI) procedures in individuals with ST-segment elevation myocardial infarction (STEMI) [15]. Notably, a retrospective cohort study of 658 STEMI-PCI patients (median follow-up: 18.9 months) demonstrated via multivariable Cox regression analysis that PIV outperformed traditional inflammatory markers, such as the neutrophil-to-lymphocyte ratio (NLR), platelet-to-lymphocyte ratio (PLR), and systemic immune-inflammation index (SII), in predicting composite endpoints, including major adverse cardiovascular events (MACE) and all-cause mortality [16]. Nevertheless, existing evidence remains limited, particularly regarding data on PIV’s impact on long-term outcomes in MI survivors. This underscores the need for prospective studies to validate its clinical prognostic utility.

This study utilizes nationally representative data spanning 2001–2018 from the National Health and Nutrition Examination Survey (NHANES) to investigate the predictive value of PIV in assessing the association with both all-cause mortality and cardiovascular-related deaths in individuals following MI, providing new evidence for clinical decision-making.

## 2. Materials and Methods

### 2.1. Data Source

**Figure 1:**
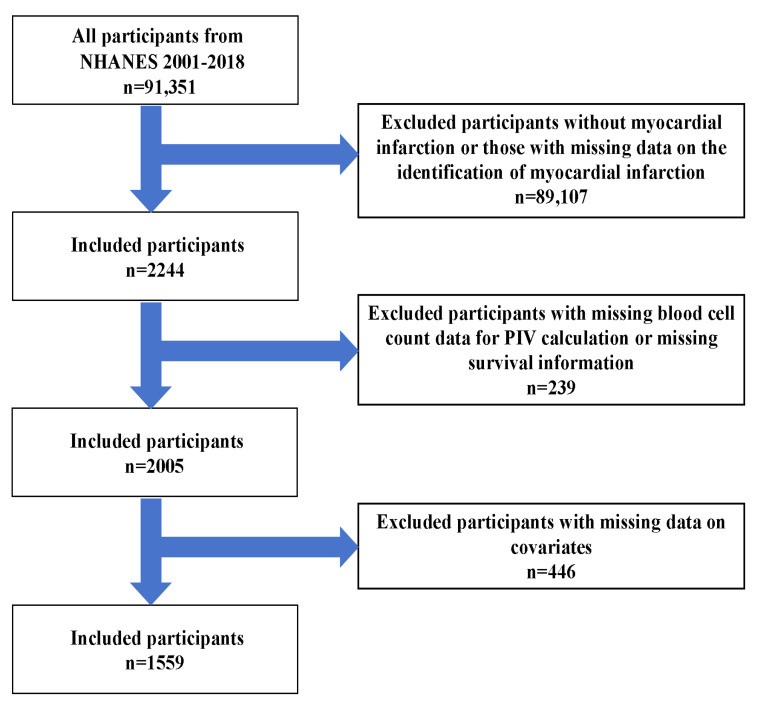
Flowchart of participants’ inclusion and exclusion criteria from NHANES 2001–2018.

This study leveraged nine consecutive NHANES survey cycles (2001–2018) obtained through the CDC’s National Center for Health Statistics (NCHS) online repository (https://wwwn.cdc.gov/nchs/nhanes/, accessed on 10 July 2025). NHANES, a core surveillance program of the NCHS, employs a multistage probability sampling design to systematically collect health and nutritional data from the non-institutionalized U.S. population, ensuring national representativeness [17]. During the 2001–2018 survey period, NHANES enrolled 91,351 participants. Screening excluded individuals who: (1) were without MI or those with missing data on the identification of MI; (2) had missing blood cell count data for PIV calculation; (3) lacked survival information; or (4) had incomplete covariate data. Ultimately, 1559 MI survivors were included in the analysis. Detailed inclusion and exclusion criteria are illustrated in Figure 1.

### 2.2. Identification of MI Survivors

MI survivors were identified using a validated self-report instrument. Participants responding affirmatively to the diagnostic inquiry, “Has a doctor or other health professional ever diagnosed you with a heart attack (also called MI)?” were categorized as MI survivors [18]. The use of self-reported physician-diagnosed MI in NHANES has been common in prior epidemiologic studies, with multiple studies confirming the reliability of this assessment method [19,20,21,22]. However, it should be acknowledged that self-reported information may be influenced by recall bias, potentially affecting the study outcomes.

### 2.3. Calculation and Logarithmic Transformation of the PIV

Blood specimens were obtained from participants at NHANES Mobile Examination Centers (MECs), which are standardized mobile units operated by the NCHS that travel across the United States to collect health data and biological samples from a nationally representative population. Across the 2001–2018 survey cycles, hematological profiling—including quantification of neutrophils, lymphocytes, monocytes, and platelets was performed on-site using the Beckman Coulter automated blood analyzer (Beckman Coulter, Inc., Brea, CA, USA). PIV was calculated using the established formula: [Neutrophil count (×10^3^ cells/µL)] × [Platelet count (×10^3^ cells/µL)] × [Monocyte count (×10^3^ cells/µL)]/[Lymphocyte count (×10^3^ cells/µL)]. All laboratory measurements followed standardized analytical procedures under Clinical Laboratory Improvement Amendments (CLIA)-certified quality control protocols. In the NHANES survey, participants identified as MI survivors were those who responded affirmatively to the standardized question. This definition inherently includes individuals with a prior, physician-confirmed diagnosis of MI who had survived the acute episode, received appropriate treatment, and were living in the community at the time of the survey. As a result, all blood specimens used in our study were collected during the recovery/chronic phase of MI, rather than in the acute or early subacute period. Every PIV in our study was calculated from blood cell counts measured in MI survivors during their recovery phase, ensuring a consistent time frame for all participants. To normalize the distribution characteristics of the PIVs, natural logarithmic conversion was employed for subsequent statistical analyses.

### 2.4. Mortality and Follow-Up Assessment

This study defined the primary endpoint as all-cause mortality and the secondary endpoint as cardiovascular-specific mortality, encompassing two categories of fatal events: (1) Cardiovascular-related deaths: Identified using ICD-10 codes I00–I09, I11, I13, and I20–I51. (2) Cerebrovascular-related deaths: Identified using ICD-10 codes I60–I69. Mortality outcomes were ascertained through probabilistic linkage with the National Death Index (NDI), with death certificates reviewed to validate cause-of-death classification. Follow-up duration was calculated from the date of baseline assessment at the NHANES Mobile Examination Center (MEC) until the earliest of: (1) confirmed death date (via NDI records) or (2) 31 December 2019 (study cutoff date).

### 2.5. Covariate Assessment

The statistical models incorporated adjustments for multiple demographic and clinical parameters as covariates, such as gender, age, race, education level, marital status, poverty-to-income ratio (PIR), and BMI (body mass index). Additional covariates comprised smoking status (categorized per CDC guidelines: never/former [≥100-lifetime cigarettes with cessation]/current [ongoing use after ≥100 cigarettes]), drinking status (regular consumption defined as ≥12 drinks within the preceding year), diabetes status (diagnosis based on fasting glucose ≥ 126 mg/dL, HbA1c ≥ 6.5%, self-reported history, or hypoglycemic agent use) [23], hypertension (self-reported diagnosis, antihypertensive therapy, or three sequential measurements blood pressure ≥ 140/90 mmHg) [24], Hypercholesterolemia (self-reported history of hypercholesterolemia, total cholesterol level ≥ 200 mg/dL, taking medication to lower cholesterol) [25], other comorbidities (heart failure, stroke, and cancer everified through validated questionnaires), and renal function metrics quantified via the CKD-EPI creatinine equation [26]. This formula integrates serum creatinine values with adjustments for age, sex, and ethnicity to estimate glomerular filtration rate (eGFR) with enhanced precision.

### 2.6. Statistical Analysis

To account for NHANES’s multistage stratified cluster probability sampling design, analyses incorporated sampling weights per NCHS guidelines. Data from the 2001–2018 cycles were merged, and the Mobile Examination Center two-year sample weight (WTMEC2YR) was applied to generate integrated weights for cross-cycle analyses. Baseline characteristics were stratified by survival status (alive/deceased): categorical data were expressed in proportions (%) while continuous measurements presented mean values with standard deviations. Statistical comparisons between groups employed parametric *t*-tests (Student’s method), nonparametric Wilcoxon tests, exact probability tests (Fisher’s), and chi-square analyses, selected based on data characteristics. Weighted Cox proportional hazard models with multivariable adjustment were constructed to assess the relationships of log-transformed PIV (LnPIV) with mortality risk. Results were expressed as Hazard Ratios (HRs) and 95% Confidence Interval (95%CI) across four hierarchical models: model1: No covariate adjustment; model 2: Demographic/socioeconomic confounders (age, gender, race, education level, PIR, marital status); model3: Metabolic/behavioral factors (BMI, smoking status, drinking status), clinical comorbidities (diabetes mellitus, hypertension, hypercholesterolemia, heart failure, stroke, cancer), and eGFR; model 4: all aforementioned covariates combined. To assess dose–response relationships, LnPIV was categorized into quartiles (Q1–Q4, Q1 as reference), with trend tests evaluating statistical significance. Restricted cubic spline (RCS) curves with four knots explored potential non-linear associations between LnPIV and mortality, identifying inflection points. Survival curves were plotted via the Kaplan–Meier approach, with group differences assessed through log-rank tests. Subgroup analyses stratified by key variables assessed heterogeneity and interaction effects via stratified Cox models. Time-dependent ROC curves alongside AUC (area under the curve) metrics were employed to quantify LnPIV’s predictive capacity for mortality across defined time intervals. Analytical procedures were conducted with R (version 4.2.2) and EmpowerStats (v4.0). A two-tailed *p* < 0.05 indicated statistical significance.

## 3. Results

### 3.1. Baseline Characteristics

Table 1 summarizes the baseline characteristics of all participants. After applying inclusion and exclusion criteria, 1559 MI survivors were included in the analysis, comprising 1051 males and 508 females. Over a median follow-up period of 75 months, 675 participants experienced mortality events. Compared to survivors, deceased participants were older and exhibited: lower educational attainment, PIR, BMI, eGFR, and proportion of alcohol consumers, higher rates of smoking, hypertension, diabetes mellitus, heart failure, stroke, and cancer, elevated PIV and LnPIV. Significant differences were also observed in racial composition and marital status between the two groups.

### 3.2. Association Between LnPIV and All-Cause and Cardiovascular Mortality in MI Survivors

During the median follow-up period of 75 months, 675 deaths were recorded, including 291 cardiovascular-specific deaths. Table 2 presents the associations between LnPIV and mortality outcomes. Multivariable Cox proportional hazard models with weighting were utilized to evaluate independent relationships. In continuous-scale evaluations of log-transformed PIV (LnPIV), the unadjusted model (Model 1) revealed that each standard unit increase in LnPIV was linked to a 40% higher risk of all-cause mortality (HR = 1.40, 95%CI: 1.19–1.64) and a 49% increased cardiovascular mortality (HR = 1.49, 95%CI: 1.17–1.89). After accounting for sociodemographic factors (Model 2), the effect estimates decreased to HR = 1.21 (95%CI: 1.02–1.44) for all-cause mortality and HR = 1.30 (95%CI: 1.01–1.67) for cardiovascular mortality. When we further adjusted only for metabolic behavioral factors, clinical comorbidities, and eGFR (Model 3), the associations remained significant (HR = 1.39, 95%CI: 1.17–1.64 for all-cause mortality; HR = 1.50, 95%CI: 1.17–1.91 for cardiovascular mortality), indicating that the relationship persisted even after adjusting for these health-related factors. However, in the fully adjusted model (Model 4), which incorporated all covariates, the HR were reduced to 1.17 (95%CI: 0.99–1.39) for all-cause mortality and 1.26 (95%CI: 0.97–1.64) for cardiovascular mortality, and both lost statistical significance (*p* > 0.05).

When analyzing LnPIV quartiles, the unadjusted model showed progressive risk increments: compared with the lowest quartile (Q1), higher quartiles demonstrated HRs of 0.98 (95%CI: 0.68–1.41) for Q2, 1.17 (95%CI: 0.86–1.60) for Q3, and 1.83 (95%CI: 1.35–2.47) for Q4 regarding all-cause mortality. Cardiovascular mortality exhibited comparable patterns across quartiles. Adjusting only for sociodemographic or clinical/metabolic factors separately (Models 2 and 3) attenuated but generally preserved statistical significance for the highest quartiles. Following comprehensive adjustment (Model 4), the modified HRs were attenuated to 0.87 (0.61–1.25), 0.83 (0.61–1.15), and 1.29 (0.94–1.77) for all-cause mortality quartiles, and 1.04 (0.67–1.62), 1.00 (0.63–1.60), and 1.41 (0.88–2.27) for cardiovascular mortality quartiles.

Trend analyses demonstrated significant dose–response relationships for both mortality outcomes in Model 1 (*p*-trend < 0.001 for all-cause; *p*-trend = 0.001 for cardiovascular). However, these linear associations were substantially attenuated in the fully adjusted model (Model 4), with non-significant *p*-trend values of 0.077 and 0.162, respectively.

### 3.3. RCS Analysis

**Figure 2:**
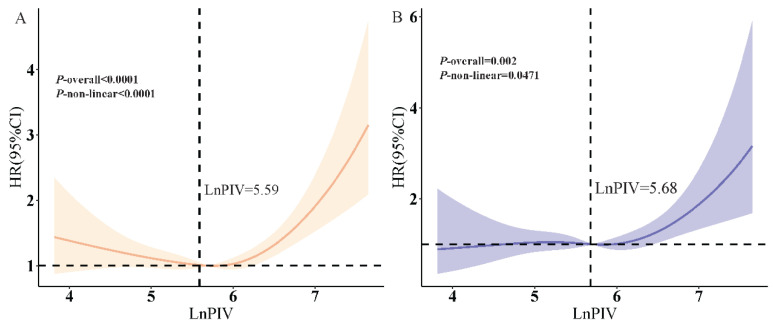
RCS curves depicting threshold effects of LnPIV on (**A**) all-cause mortality (*p* for non-linearity < 0.0001) and (**B**) cardiovascular mortality (*p* for non-linearity = 0.0471). The vertical dotted lines indicate the threshold inflection points of LnPIV (5.59 for all-cause mortality and 5.68 for cardiovascular mortality), and the horizontal dotted line denotes the reference line at HR = 1.

To investigate potential non-linear associations between LnPIV and mortality risk, RCS analysis was conducted using four predefined knot positions (5th, 35th, 65th, 95th percentiles), derived from covariates in the fully adjusted model (Model 3). Analyses revealed significant non-linear associations between LnPIV and both all-cause mortality (*p* for non-linearity < 0.0001) and cardiovascular mortality (*p* for non-linearity = 0.0471) after comprehensive adjustment (Figure 2). Two-segment Cox proportional hazards models were utilized to confirm these relationships. Critical threshold identification showed inflection points at LnPIV = 5.59 for all-cause mortality and LnPIV = 5.68 for cardiovascular mortality (Table 3). Below 5.59, no mortality risk elevation was observed with LnPIV variation (HR = 0.76, 95% CI: 0.51–1.13), whereas each unit increase above this threshold raised all-cause mortality risk by 85% (HR = 1.85, 95% CI: 1.49–2.28). Similarly, cardiovascular mortality risk showed no significant correlation with LnPIV below 5.68 (HR = 0.98, 95% CI: 0.63–1.52) but surged significantly beyond this point (HR = 1.77, 95% CI: 1.20–2.63). Threshold validity was confirmed by likelihood ratio tests (all *p* < 0.05).

### 3.4. Kaplan–Meier Survival Analysis

**Figure 3:**
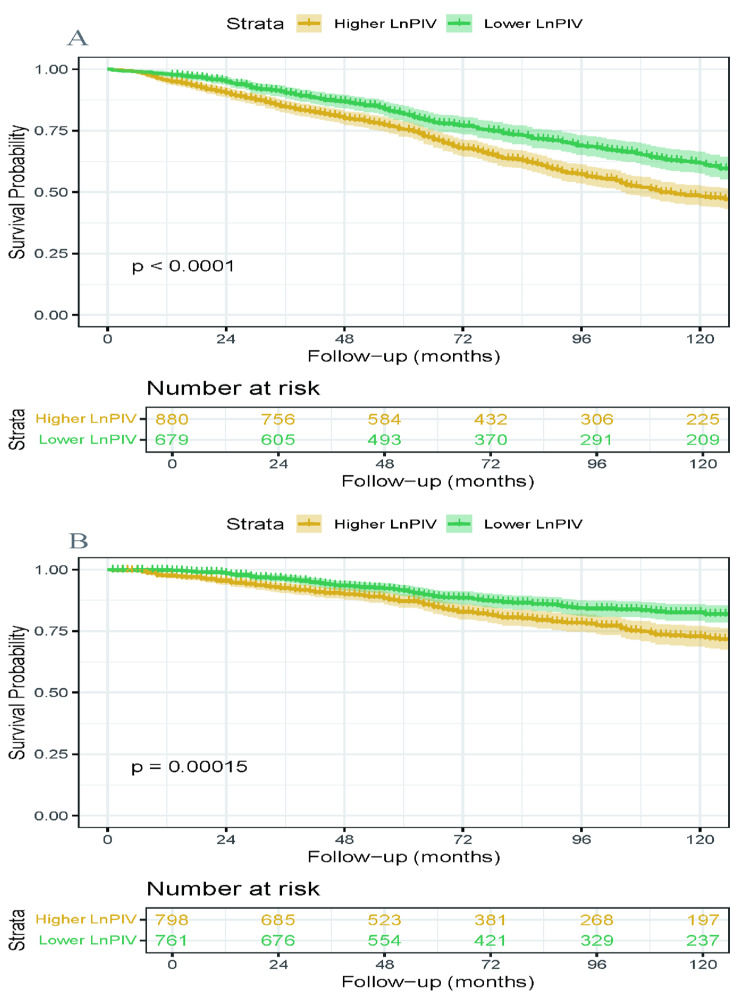
Kaplan–Meier survival curves stratified by LnPIV thresholds. Cumulative survival probabilities for all-cause mortality (**A**) threshold: LnPIV = 5.59 and cardiovascular mortality (**B**) threshold: LnPIV = 5.68.

**Figure 4:**
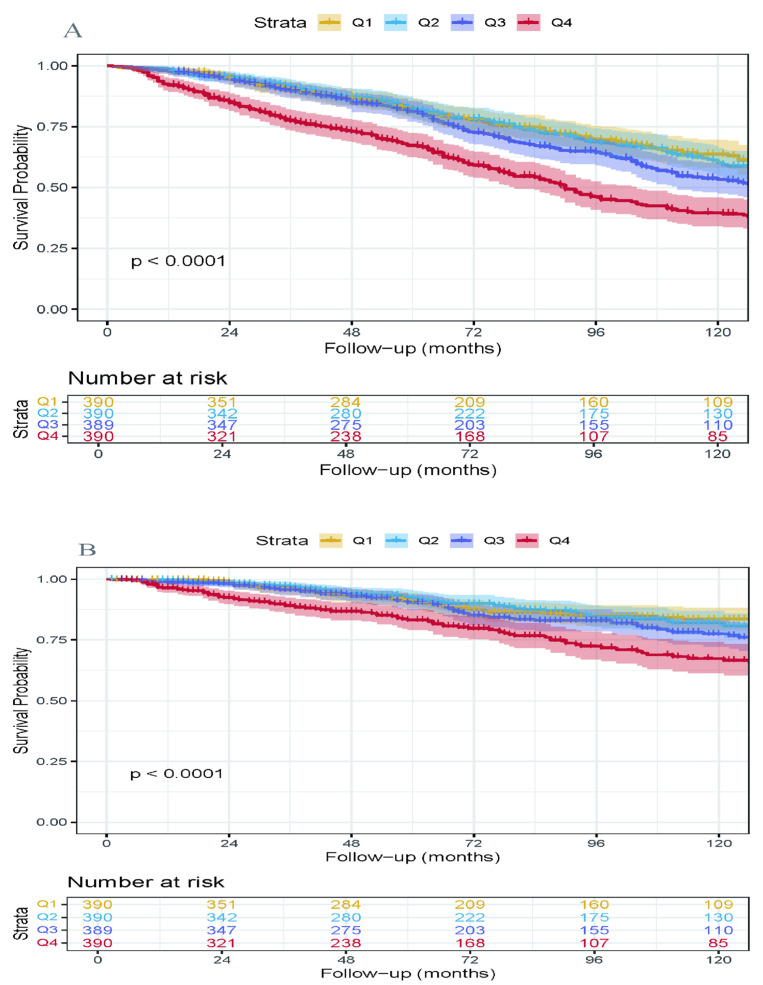
Kaplan–Meier survival curves illustrating cumulative survival probabilities all-cause mortality (**A**) and cardiovascular mortality (**B**) across quartile levels of the LnPIV.

Based on the threshold determined by RCS, participants were categorized into higher/lower LnPIV groups. For all-cause mortality, using LnPIV = 5.59 as the cutoff, the higher LnPIV group (≥5.59) and lower LnPIV group (<5.59) were established; for cardiovascular mortality, the cutoff was LnPIV = 5.68, defining higher LnPIV (≥5.68) and lower LnPIV (<5.68) groups. The Kaplan–Meier survival curves (Figure 3) demonstrated that the higher LnPIV group exhibited significantly lower cumulative survival rates in both all-cause mortality (*p* for log-rank test < 0.0001) and cardiovascular mortality (*p* for log-rank test = 0.00015). Figure 4 presented the Kaplan–Meier curves illustrating all-cause mortality and cardiovascular mortality across quartile levels of the LnPIV (Q1–Q4). They demonstrated that in the survivors of MI, the highest LnPIV quartiles (Q4 vs. Q1) had significantly lower overall survival and cardiovascular-specific survival probabilities (*p* for log-rank test < 0.0001).

### 3.5. Subgroup Analysis

**Figure 5:**
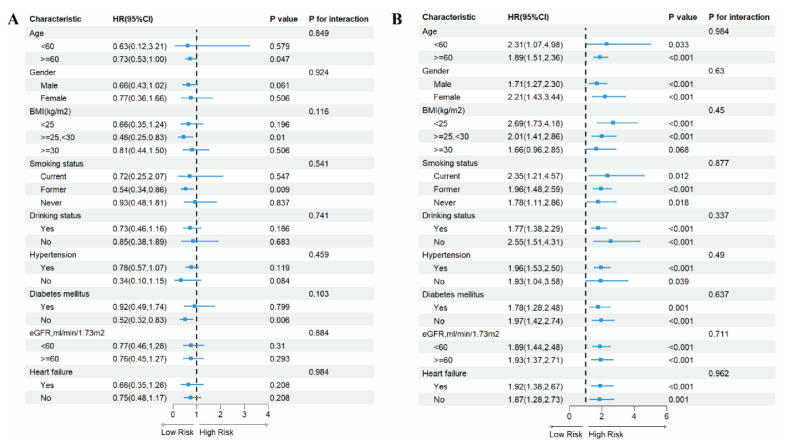
Subgroup analyses of the associations between LnPIV and all-cause mortality. (**A**) Associations between LnPIV and all-cause mortality among participants with LnPIV < 5.59. (**B**) Associations between LnPIV and all-cause mortality among participants with LnPIV ≥ 5.59. All models were adjusted for age, gender, race, education level, PIR, marital status, BMI, smoking status, drinking status, diabetes mellitus, hypertension, hypercholesterolemia, heart failure, stroke, cancer, and eGFR, except for the subgroup variable.

**Figure 6:**
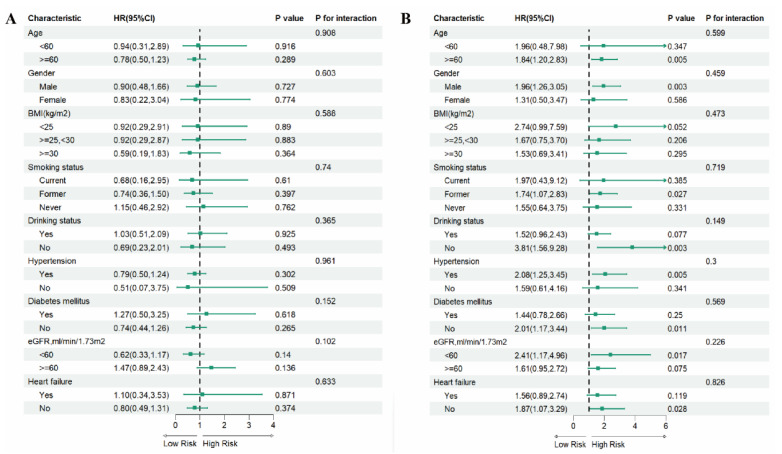
Subgroup analyses of the associations between LnPIV and cardiovascular mortality. (**A**) Associations between LnPIV and cardiovascular mortality among participants with LnPIV < 5.68. (**B**) Associations between LnPIV and cardiovascular mortality among participants with LnPIV ≥ 5.68. All models were adjusted for age, gender, race, education level, PIR, marital status, BMI, smoking status, drinking status, diabetes mellitus, hypertension, hypercholesterolemia, heart failure, stroke, cancer, and eGFR, except for the subgroup variable.

To evaluate the effect size heterogeneity of the association between LnPIV and mortality risks in MI survivors, this study employed stratified Cox proportional hazards models for subgroup interaction analyses across different subgroups (Figure 5 and Figure 6). Results demonstrated: Among participants with LnPIV < 5.59 (Figure 5A), Each unit increase in LnPIV was inversely associated with all-cause mortality risk (HR < 1) in age ≥ 60, overweight, former smokers, non-diabetic subgroups, while no association was observed in other subgroups. For participants with LnPIV ≥ 5.59 (Figure 5B), increased LnPIV levels elevated all-cause mortality risk across most subgroups, though interaction tests lacked statistical significance (*p* for interaction > 0.05). Similarly, in cardiovascular mortality analysis for LnPIV < 5.68 (Figure 6A), no association was observed between cardiovascular mortality risk and changes in LnPIV, with no significant interaction effects detected across all subgroups. Among participants with LnPIV ≥ 5.68 (Figure 6B), elevated LnPIV levels increased cardiovascular mortality risk in subgroups of age ≥ 60, male, former smokers, non-drinkers, hypertensive, non-diabetic, eGFR < 60, and non-heart failure subgroups, with no statistically significant interactions detected (*p* for interaction > 0.05).

### 3.6. ROC Analysis

**Figure 7:**
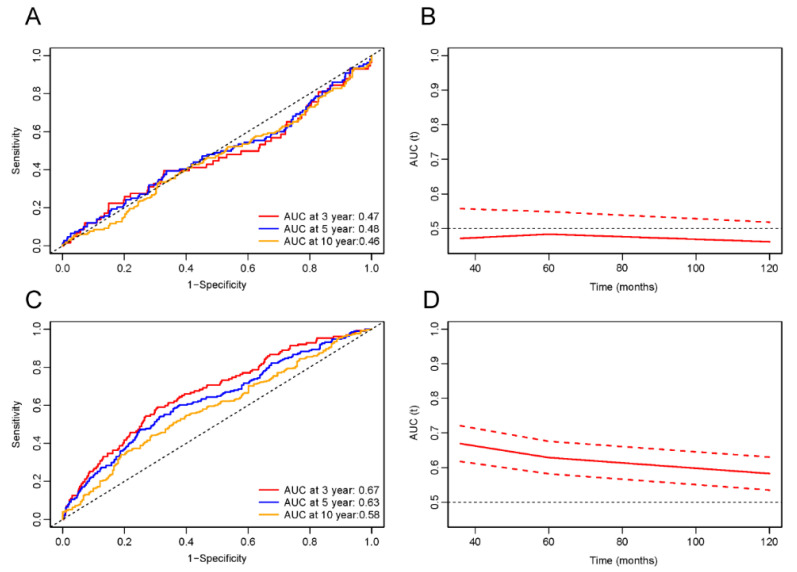
Time-dependent ROC curves and AUC values of LnPIV for predicting all-cause mortality. (**A**) Time-dependent ROC curves when LnPIV < 5.59. (**B**) Time-dependent AUC values (solid line) with 95% confidence bands (dashed lines) when LnPIV < 5.59. (**C**) Time-dependent ROC curves when LnPIV ≥ 5.59. (**D**) Time-dependent AUC values (solid line) with 95% confidence bands (dashed lines) when LnPIV ≥ 5.59.

**Figure 8:**
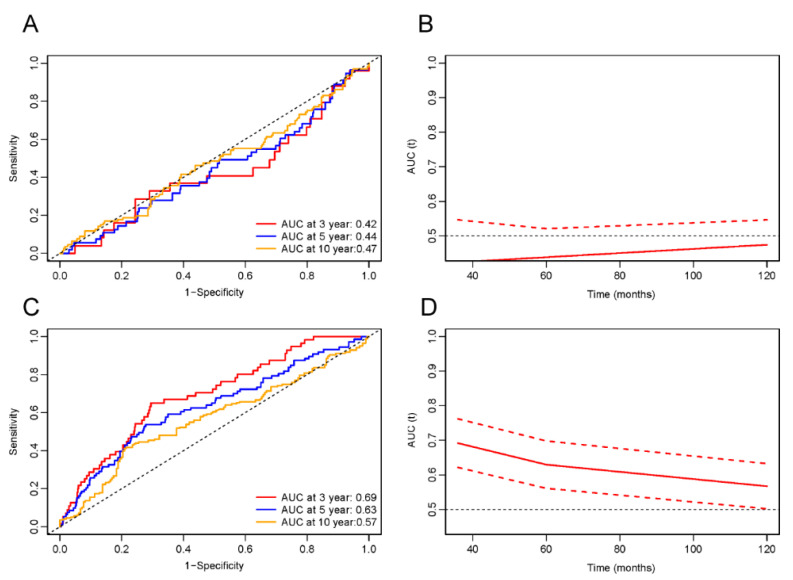
Time-dependent ROC curves and AUC values of LnPIV for predicting cardiovascular mortality. (**A**) Time-dependent ROC curves when LnPIV < 5.68. (**B**) Time-dependent AUC values (solid line) with 95% confidence bands (dashed lines) when LnPIV < 5.68. (**C**) Time-dependent ROC curves when LnPIV ≥ 5.68. (**D**) Time-dependent AUC values (solid line) with 95% confidence bands (dashed lines) when LnPIV ≥ 5.68.

The predictive capability of LnPIV for all-cause and cardiovascular mortality in MI survivors was evaluated through time-dependent ROC curve analysis, revealing significant threshold-dependent effects (Figure 7 and Figure 8). When LnPIV < 5.59 (Figure 7A), the ROC demonstrated AUC performance in assessing all-cause mortality over 3-, 5-, and 10-year intervals, yielding corresponding values of 0.47, 0.48, and 0.46. indicating no clinical predictive value. However, when LnPIV ≥ 5.59 (Figure 7C), the AUC values significantly improved to 0.67, 0.63, and 0.58, with optimal 3-year predictive performance. Similarly, for cardiovascular mortality prediction, the AUC values were 0.42, 0.44, and 0.47 when LnPIV < 5.68 (Figure 8A), but increased to 0.69, 0.63, and 0.57 above this threshold (Figure 8C). Time-dependent AUC curves confirmed that LnPIV ≥ 5.59 effectively predicts both short-term and long-term all-cause mortality (Figure 7B,D), while LnPIV ≥ 5.68 demonstrates effective predictive capability for short-term and long-term cardiovascular mortality (Figure 8B,D).

## 4. Discussion

This study analyzed data from NHANES 2001–2018 and revealed a non-linear association between LnPIV and all-cause as well as cardiovascular mortality among MI survivors. It also identified predictive thresholds for LnPIV (all-cause mortality: LnPIV ≥ 5.59; cardiovascular mortality: LnPIV ≥ 5.68). Unlike previous studies that primarily focused on short-term outcomes in specific clinical populations, this study evaluated the long-term prognostic value of PIV in a nationally representative cohort of MI survivors with extended follow-up. Moreover, the identification of clear threshold effects through RCS analysis introduced clinically actionable cutoffs, which could guide risk stratification and individualized inflammatory management. These findings provide critical evidence supporting PIV as a potential biomarker for long-term prognosis after MI, while also contributing to a deeper understanding of the mechanistic role of inflammation in cardiovascular disease outcomes.

Inflammation plays a central role in the pathophysiology of MI onset and progression [27]. Inflammatory cells and their secreted factors promote atherosclerotic plaques’ formation, progression, and rupture, thereby triggering MI [28]. During the acute phase of MI, necrotic cardiomyocytes trigger a robust inflammatory response, recruiting inflammatory cells to clear necrotic tissue [29]. In the repair phase, inflammation facilitates tissue healing, but excessive inflammation may lead to adverse ventricular remodeling and cardiac dysfunction [30,31]. Neutrophils are among the first immune cells to infiltrate the infarcted area [32]. In the acute phase, neutrophils release reactive oxygen species (ROS) and pro-inflammatory cytokines such as IL-1β and TNF-α, and secrete chemokines including CXCL1 to recruit monocytes [33,34]. During the repair phase, neutrophils can shift from an N1 to an N2 phenotype, promoting macrophage polarization toward the reparative M2 type and suppressing inflammation [35]. However, persistently activated neutrophils and their cytotoxic products may exacerbate ventricular remodeling and myocardial fibrosis [36]. Clinical studies indicate that elevated peripheral neutrophil levels are strongly associated with poor long-term outcomes and increased mortality risk in patients with acute coronary syndrome [37]. Monocytes migrate to the infarct zone during the acute phase and differentiate into M1 macrophages, releasing proteases and pro-inflammatory cytokines such as IL-6, which aggravate myocardial injury [38]. In the repair phase, monocytes transition to the M2 phenotype, promoting collagen deposition and scar formation to maintain cardiac structural stability [39]. Elevated monocyte levels in MI survivors may reflect heightened inflammatory activity around atherosclerotic plaques, increasing the risk of plaque rupture and recurrent MI [40]. Beyond their role in coagulation, platelets contribute to inflammation by releasing pro-inflammatory mediators and interacting with other immune cells, thereby increasing the risk of coronary adverse events such as in-stent restenosis or non-target lesion progression [41]. Previous cohort studies have shown that elevated baseline platelet counts significantly increase two-year mortality risk in patients with prior acute MI [42]. T lymphocytes, particularly Th1 and Th17 subsets, are activated and recruited to the infarcted area during the acute phase, releasing inflammatory cytokines such as IFN-γ and TNF-α. B lymphocytes indirectly modulate inflammation through antibody production and interactions with other immune cells [43,44,45]. During the repair phase, Treg and Th2 cells secrete anti-inflammatory cytokines such as IL-10 and TGF-β, promoting fibrosis and scar stabilization while clearing tissue debris and restoring immune homeostasis to prevent maladaptive remodeling [46,47]. A significant reduction in peripheral blood lymphocytes in post-MI patients may indicate an immunosuppressive state, impairing tissue repair, exacerbating adverse cardiac remodeling, and worsening long-term prognosis [48,49]. Thus, the inflammatory status in post-MI patients is closely linked to clinical outcomes, necessitating a comprehensive assessment.

The PIV integrates neutrophil, platelet, monocyte, and lymphocyte counts, providing a comprehensive assessment of systemic immune-inflammatory burden. This study exclusively enrolled post-MI patients in the recovery phase. Results revealed a significant non-linear dose–response relationship between LnPIV and mortality risk. RCS analysis demonstrated that below the thresholds of 5.59 for all-cause mortality and 5.68 for cardiovascular mortality, elevated LnPIV did not significantly increase mortality risk, with some subgroups even exhibiting a protective effect (HR < 1). However, beyond these thresholds, higher LnPIV was strongly associated with increased risks of both all-cause and cardiovascular mortality. This phenomenon reflects the “double-edged sword” effect of inflammation, moderate inflammation facilitates tissue debris clearance and repair, whereas excessive inflammation may induce secondary myocardial injury, plaque destabilization, and ventricular remodeling through mechanisms such as ROS release, pro-inflammatory cytokine production, and inflammatory cell infiltration [50,51]. Elevated neutrophils, monocytes, and platelets may synergistically drive this process, while lymphopenia further impairs immunoregulatory capacity, amplifying inflammatory damage. These factors likely underline the non-linear association between LnPIV and mortality risk in MI survivors, though precise mechanisms warrant further investigation. Moreover, the threshold effect of PIV offers novel insights for personalized risk management. For post-MI patients exceeding the thresholds, intensified anti-inflammatory therapies (e.g., colchicine, IL-1β antagonists) and enhanced monitoring could be considered to improve prognosis [52,53]. Notably, as PIV is derived from routine complete blood count (CBC) parameters, it is cost-effective and readily accessible, making it particularly suitable for large-scale application in resource-limited settings. Sex-specific differences in the inflammatory response and clinical outcomes after MI have gained increasing attention. Emerging evidence suggests that females may exhibit a heightened inflammatory activation profile compared to males, yet experience distinct post-MI remodeling pathways and outcome trajectories [54]. While our subgroup analysis did not detect statistically significant interactions by sex, slight variations in effect estimates between males and females were observed, warranting further investigation in sex-stratified cohorts to determine whether tailored PIV thresholds or intervention strategies may be appropriate. It is important to note that the predictive performance of LnPIV, as measured by time-dependent AUC, showed a gradual decline during long-term follow-up. This pattern is consistent with prior biomarker research [49,55] and may be attributed to the fact that a single baseline inflammatory measurement most strongly reflects short- to mid-term risk. As time progresses, additional factors, such as changes in clinical status, comorbidities, treatments, and lifestyle, may increasingly influence mortality, thereby diluting the prognostic contribution of baseline LnPIV. Furthermore, the inflammatory response may play a more prominent role in early adverse events following MI, whereas later outcomes could be driven by structural remodeling, neurohormonal activation, or renal dysfunction, which are not captured by LnPIV. Despite this temporal attenuation, LnPIV maintained meaningful discriminative capacity over time, supporting its value as a baseline risk stratification tool.

This study has several limitations. First, the observational nature NHANES data cannot fully exclude residual confounding. Second, the identification of MI survivors was based on self-reported physician diagnosis, which may introduce recall and selection bias. Third, PIV calculation relies on single baseline measurements, failing to capture dynamic inflammatory changes. Fourth, the predominant inclusion of U.S. residents limits external validity, and the generalizability of our findings across different ethnicities and healthcare systems requires further verification. Finally, mechanistic interpretations remain constrained to statistical associations, underscoring the need for experimental and longitudinal studies to elucidate the specific biological pathways through which PIV contributes to adverse outcomes.

## 5. Conclusions

Our findings suggest a non-linear association between LnPIV and fatal outcomes in MI survivors, potentially quantifying clinically relevant predictive values for adverse prognosis. If validated in future mechanistic and clinical studies, this association could offer a potential tool for inflammatory monitoring and risk stratification and may inform the development of precision interventions targeting hyperinflammatory responses.

## Figures and Tables

**Table 1:** Baseline characteristics of MI survivors.

Characteristic	Total Participants (*n* = 1559)	Surviving Participants (*n* = 884)	Dead Participants (*n* = 675)	*p*-Value
Age, years	65.01 ± 12.28	61.59 ± 12.35	70.82 ± 9.73	<0.0001
Gender, *n* (%)				0.4602
male	1051 (63.69)	575 (63.00)	476 (64.86)	
female	508 (36.31)	309 (37.00)	199 (35.14)	
Race, *n* (%)				0.0003
Mexican American	140 (3.27)	86 (3.71)	54 (2.52)	
Other Hispanic	101 (3.36)	76 (4.33)	25 (1.73)	
Non-Hispanic White	960 (79.05)	484 (75.78)	476 (84.59)	
Non-Hispanic Black	278 (9.08)	178 (9.71)	100 (8.01)	
Other Race	80 (5.23)	60 (6.46)	20 (3.15)	
Education Level, *n* (%)				<0.0001
Under high school	535 (25.51)	258 (20.68)	277 (33.72)	
High school or equivalent	388 (28.57)	218 (28.27)	170 (29.09)	
Above high school	636 (45.91)	408 (51.05)	228 (37.19)	
PIR	2.66 ± 1.61	2.84 ± 1.68	2.34 ± 1.42	<0.0001
Marital status, *n* (%)				
Married/Living with a partner	893 (61.48)	535 (65.55)	358 (54.57)	
Never married	94 (5.44)	60 (5.95)	34 (4.58)	
Widowed/Divorced/Separated	572 (33.08)	289 (28.50)	283 (40.85)	
BMI, kg/m^2^	30.32 ± 6.81	30.87 ± 6.83	29.39 ± 6.67	<0.0001
Hypercholesterolemia, *n* (%)				0.1065
Yes	1190 (77.46)	695 (78.78)	495 (75.24)	
No	369 (22.54)	189 (21.22)	180 (24.76)	
Smoking, *n* (%)				0.0001
never	496 (31.08)	299 (32.94)	197 (27.93)	
former	698 (44.45)	352 (40.39)	346 (51.35)	
current	365 (24.46)	233 (26.66)	132 (20.73)	
Drinking, *n* (%)				0.0017
Yes	1155 (76.33)	679 (78.92)	476 (71.94)	
No	404 (23.67)	205 (21.08)	199 (28.06)	
Hypertension, *n* (%)				0.0012
Yes	1176 (72.18)	646 (69.36)	530 (76.97)	
No	383 (27.82)	238 (30.64)	145 (23.03)	
Diabetes mellitus, *n* (%)				0.0003
Yes	637 (38.44)	344 (35.06)	293 (44.18)	
No	922 (61.56)	540 (64.94)	382 (55.82)	
Heart failure, *n* (%)				<0.0001
Yes	520 (30.93)	251(25.38)	269 (40.36)	
No	1039 (69.07)	633 (74.62)	406 (59.64)	
Stroke, *n* (%)				0.0073
Yes	275 (16.19)	138 (14.27)	137 (19.45)	
No	1284 (83.81)	746 (85.73)	538 (80.55)	
Cancer, *n* (%)				<0.0001
Yes	332 (21.49)	154 (17.67)	178 (27.98)	
No	1227 (78.51)	730 (82.33)	497 (72.02)	
eGFR (mL/min/1.73 m^2^)				<0.0001
<60	510 (28.25)	196 (19.71)	314 (42.75)	
≥60	1049 (71.75)	688 (80.29)	361 (57.25)	
PIV	380.33 ± 320.41	343.49 ± 256.88	442.89 ± 398.40	<0.0001
LnPIV	5.69 ± 0.71	5.62 ± 0.67	5.81 ± 0.75	<0.0001

Categorical variables are depicted with frequencies and percentages, while continuous variables are shown as mean ± SD.

**Table 2:** Multivariable-adjusted associations between LnPIV and mortality.

LnPIV	Model 1		Model 2		Model 3		Model 4	
	HR (95%CI)	*p*	HR (95%CI)	*p*	HR (95%CI)	*p*	HR (95%CI)	*p*
All-cause mortality								
Continuous	1.40 (1.19, 1.64)	<0.001	1.21 (1.02, 1.44)	0.026	1.39 (1.17, 1.64)	<0.001	1.17 (0.99, 1.39)	0.070
Categorical								
Q1 (2.09–5.23)	Reference		Reference		Reference		Reference	
Q2 (5.23–5.71)	0.98 (0.68, 1.41)	0.913	0.90 (0.65, 1.24)	0.518	0.94 (0.63, 1.40)	0.757	0.87 (0.61, 1.25)	0.445
Q3 (5.71–6.13)	1.17 (0.86, 1.60)	0.327	0.86 (0.62, 1.19)	0.373	1.15 (0.84, 1.58)	0.391	0.83 (0.61, 1.15)	0.267
Q4 (6.13–8.20)	1.83 (1.35, 2.47)	<0.001	1.39 (1.04, 1.88)	0.029	1.69 (1.22, 2.34)	0.002	1.29 (0.94, 1.77)	0.112
*p* for trend	<0.001		0.023		<0.001		0.077	
Cardiovascular mortality								
Continuous	1.49 (1.17, 1.89)	0.001	1.30 (1.01, 1.67)	0.043	1.50 (1.17, 1.91)	0.001	1.26 (0.97, 1.64)	0.083
Categorical								
Q1 (2.09–5.23)	Reference		Reference		Reference		Reference	
Q2 (5.23–5.71)	1.16 (0.75, 1.79)	0.499	1.07 (0.70, 1.65)	0.749	1.14 (0.72, 1.79)	0.580	1.04 (0.67, 1.62)	0.858
Q3 (5.71–6.13)	1.36 (0.86, 2.16)	0.188	1.02 (0.64, 1.62)	0.946	1.40 (0.88, 2.22)	0.157	1.00 (0.63, 1.60)	0.991
Q4 (6.13–8.20)	2.01 (1.31, 3.08)	0.001	1.51 (0.96, 2.39)	0.074	1.90 (1.22, 2.96)	0.005	1.41 (0.88, 2.27)	0.151
*p* for trend	0.001		0.087		0.003		0.162	

HR: hazard ratio; 95%CI: 95% Confidence Interval; Model 1: without any covariate adjustments; Model 2: adjusted for age, gender, race, education level, PIR, marital status; Model 3: adjusted for BMI, smoking status, drinking status, diabetes mellitus, hypertension, heart failure, stroke, cancer, hypercholesterolemia, eGFR; Model 4: adjusted for age, gender, race, education level, PIR, marital status, BMI, smoking status, drinking status, diabetes mellitus, hypertension, heart failure, stroke, cancer, hypercholesterolemia, eGFR.

**Table 3:** Threshold effect analysis of LnPIV on mortality outcomes.

	Adjusted HR (95%CI), *p* Value
All-cause mortality	
Total	1.17 (0.99, 1.39), 0.070
Cutoff value	5.59
LnPIV < 5.59	0.76 (0.51, 1.13), 0.176
LnPIV ≥ 5.59	1.85 (1.49, 2.28), <0.001
*p* for Log-likelihood ratio	<0.001
Cardiovascular mortality	
Total	1.26 (0.97, 1.64), 0.083
Cutoff value	5.68
LnPIV < 5.68	0.98 (0.63, 1.52), 0.914
LnPIV ≥ 5.68	1.77 (1.20, 2.63), 0.004
*p* for Log-likelihood ratio	0.003

HR: hazard ratio; 95%CI: 95% Confidence Interval.

## Data Availability

The information presented in the article is accessible through the first author. The data can be found here: https://wwwn.cdc.gov/nchs/nhanes/, accessed on 10 July 2025.
